# Dietary intakes of vitamins A, C, and E and risk of melanoma in two cohorts of women

**DOI:** 10.1038/sj.bjc.6600882

**Published:** 2003-04-29

**Authors:** D Feskanich, W C Willett, D J Hunter, G A Colditz

**Affiliations:** 1 Channing Laboratory, Department of Medicine, Brigham and Women's Hospital and Harvard Medical School, Boston, MA 02115, USA; 2 Department of Nutrition, Harvard School of Public Health, Boston, MA 02115, USA; 3 Department of Epidemiology, Harvard School of Public Health, Boston, MA 02115, USA; 4 Harvard Center for Cancer Prevention, Harvard School of Public Health, Boston, MA 02115, USA

**Keywords:** melanoma, retinol, tocopherol, carotenoid, antioxidant, diet, women

## Abstract

Within the two Nurses' Health Study cohorts of US women, we examined whether higher intakes of vitamin C, vitamin E, retinol, or individual tocopherols or carotenoids are associated with a lower risk of melanoma. We confirmed 414 cases of invasive melanoma among over 162 000 Caucasian women aged 25–77 years during more than 1.6 million person-years of follow-up. Diet was measured every 4 years with a food frequency questionnaire and supplement use was reported every 2 years. Several measures of sun sensitivity were assessed and included in proportional hazards models. We found that vitamins A, C, E and their individual components were not associated with a lower risk of melanoma. Only retinol intake from foods plus supplements appeared protective within a subgroup of women who were otherwise at low risk based on nondietary factors (relative risk (RR)=0.39, 95% confidence interval (CI) 0.22–0.71 for ⩾1800 *vs* <400 *μ*g day^−1^, *P* for linear trend=0.01). Contrary to expectation, we observed higher risks of melanoma with greater intakes of vitamin C from food only (RR=1.43, 95% CI 1.01–2.00 for ⩾175 *vs* <90 mg day^−1^, *P* for linear trend=0.05) and a significant positive dose–response with frequency of orange juice consumption (*P*=0.008). Further research is needed to determine whether another component in foods such as orange juice may contribute to an increase in risk.

The incidence of melanoma in the US is increasing at a greater rate than that of any other cancer site (Rigel, 1997), and this is considered real and not because of earlier detection or changes in diagnostic criteria (Dennis, 1999). Excessive exposure to sunlight is the primary environmental risk factor for melanoma (Elwood and Jopson, 1997; Osterlind *et al*, 1988). Ultraviolet (UV) radiation from the sun produces free radicals that may initiate carcinogenesis through oxidative damage to DNA, and antioxidant nutrients, such as carotenoids and vitamins E and C, can neutralize the free radicals or even suppress their creation (Di Mascio *et al*, 1991; Frei, 1994). In animals, *β*-carotene and *α*-tocopherol can inhibit skin tumours induced by UV light (Black and Chan, 1975). Preformed vitamin A (i.e., retinol) is necessary for the regulation of cell differentiation and can also reduce the incidence of skin tumours in animals exposed to UV light (Levine and Meyskens, 1980; Oikarinen *et al*, 1991). Certain carotenoids (e.g., *α*-carotene, *β*-carotene, *β*-cryptoxanthin) are metabolised to retinol. These retinol precursors along with antioxidant nutrients are prominent components of fruits and vegetables and may account for the observed anticancer effects of these foods (Ziegler, 1991).

Although antioxidants and retinol appear to be beneficial for some cancers, little is known about their effect on the development of melanoma. In case–control studies, lower risks of melanoma have been associated with higher blood levels of *α*-tocopherol (Knekt *et al*, 1991), *β*-carotene (Knekt *et al*, 1991), and retinol (Breslow *et al*, 1995), and with greater dietary intakes of vitamin E (Stryker *et al*, 1990; Bain *et al*, 1993; Kirkpatrick *et al*, 1994) and *β*-carotene (Bain *et al*, 1993). Only one prospective study of diet and melanoma has been reported and no associations were found for carotenoids, retinoids, or vitamins C and E (Veierod *et al*, 1997), although the questionnaire was inadequate for accurate assessment of these nutrients.

In this prospective study within two cohorts of women, we examined the risk of melanoma by intakes of vitamin C, vitamin E and specific tocopherols, retinol, and *β*-carotene and other carotenoids.

## METHODS

### Study populations

The Nurses' Health Study (NHS) cohort consists of 121 700 female registered nurses living within 11 US states who were 30–55 years of age when they responded to the initial questionnaire in 1976. A second cohort of younger women, NHS II, was begun in 1989 with 116 671 nurses 25–42 years of age across 14 US states. Both studies were designed to examine prospectively the effects of lifestyle on risk of chronic disease. Information on individual characteristics, behaviours, and diagnosed diseases is collected with mailed questionnaires every 2 years and a response rate of at least 90% has been attained in each follow-up cycle. Deaths are confirmed through the National Death Index (Stampfer *et al*, 1984).

A baseline food frequency questionnaire (FFQ) was included in the NHS and NHS II mailings in 1980 and 1991, respectively, and diet has been reassessed at least every 4 years. In this analysis, we used the 1984 FFQ as the baseline measure for the NHS cohort since it contained 38 fruits and vegetables, significantly more than the initial 1980 questionnaire (Feskanich *et al*, 2000).

The NHS and NHS II populations for this analysis consisted of those participants who completed an FFQ at baseline and who had no previous report of cancer (except nonmelanoma skin cancer). Owing to small numbers and low risk, non-Caucasian women were also excluded. After exclusions, 73 525 women remained in the NHS study population and 88 553 women remained in the NHS II study population.

### Melanoma case confirmation

Within the study populations over the periods of follow-up for these analyses, 571 NHS women and 378 NHS II women reported a diagnosis of melanoma. Medical records were obtained for 486 (85%) of the NHS reports and for 289 (76%) of the NHS II reports; 254 (52%) and 160 (55%), respectively, were confirmed cases of invasive melanoma. These included superficial spreading and nodular types.

### Diet and supplement use

For each food on the FFQ, women reported their frequency of intake during the past year in terms of a specified serving size. Daily dietary intakes of nutrients were calculated based on the nutrient content of foods derived primarily from US Department of Agriculture sources and supplemented with data from food manufacturers and published research. Total daily nutrient intakes were calculated by adding the amounts from multivitamins and specific supplements to the intakes from food. Use of nutrient supplements was assessed on each biennial questionnaire. For multivitamins, participants provided the name brand and the number of tablets taken per week. For vitamins C, E, and A (retinol) supplements, participants specified their daily dosage. For *β*-carotene supplements, reported use was assumed to be 6000 *μ*g day^−1^.

Nutrient intakes were adjusted for total energy consumption (Willett and Stampfer, 1986) and these values were cumulatively updated in analyses to create the best estimates of long-term intake. That is, at the beginning of every 2-year follow-up cycle, each nutrient intake was calculated as the mean of all reported intakes up to that time.

Results from several validation studies in the NHS cohort indicate that nutrient intakes from the FFQ are valid for ranking subjects in diet analyses. In a comparison of the 1986 FFQ with two 1-week diet records, correlations were 0.79 for vitamin A from foods and 0.76 for vitamin C from foods (Willett, 1998). In other studies, vitamin E and *β*-carotene intakes from the FFQ predicted plasma levels of *α*-tocopherol (*r*=0.55) (Stryker *et al*, 1988) and *β*-carotene (*r*=0.31) (Michaud *et al*, 1998), respectively.

In the NHS cohort, a brief FFQ was included in the 1986 questionnaire for reporting food consumption during high school. These data were used to determine intakes earlier in life and to identify women with consistent intakes in high school and adult years.

### Covariate information

In both cohorts, age, body weight, menopausal status, parity, and use of oral contraceptives or postmenopausal hormones were assessed in each 2-year follow-up cycle. Height was reported only on the initial questionnaire and was used to calculate body mass index (BMI, kg m^−2^) in each cycle. Statistical analyses were also controlled for the following factors: skin reaction after two or more hours in the sun during childhood; number of sunburns over the lifetime (NHS) or between ages 15 and 20 (NHS II); number of moles on the left arm (NHS) or lower legs (NHS II); natural hair colour at age 21; family history (parent or sibling) of melanoma; and state of residence at cohort initiation. Information on frequency of sunscreen use was also collected, but was not used in analyses because it was unrelated to dietary intakes and was not independently associated with a decreased risk of melanoma.

### Statistical analyses

The NHS and NHS II cohorts were analysed separately. Person-time was accrued for each participant from the return date of the baseline questionnaire (mailed June 1984 for NHS; June 1991 for NHS II) until a confirmed diagnosis of melanoma, a report of another cancer, death, or end of follow-up (1 June 1998 for NHS; 1 June 1999 for NHS II). Total person-years in analysis included 956 356 from NHS and 691 784 from NHS II.

Current diet and covariate data were used to allocate person-time to the appropriate category for each variable at the beginning of each 2-year follow-up cycle. Age-adjusted incidence rates were calculated within categories of diet and supplement use, and relative risks (RRs) were then calculated as the ratio of risk in each upper category compared to the risk in the lowest, or referent, category. We used proportional hazards models to adjust the RRs for all covariate information. To examine linear trend, dietary intakes were entered into models as continuous values.

To increase precision and to obtain a single summary of the results from NHS and NHS II, a random effects model (DerSimmonian and Laird, 1986) was used to combine the RRs and to test for heterogeneity of results (*P*<0.05).

## RESULTS

Age-specific incidences of melanoma in the study populations were similar to national rates. For example, for white women 50–54 years of age, rates were 25 out of 100 000 year^−1^ in NHS and 27 out of 100 000 year^−1^ in recent US data (Ries *et al*, 2002).

Table 1

**Table 1:** Age-standardised characteristics^a^ within the low and high categories of vitamin C, vitamin E, retinol, and *β*-carotene intakes from foods plus supplements among the women in the NHS (*n*=73 432) and NHS II (*n*=88 541) study populations

	**Daily total intake**							
	**Vitamin C** (**mg**)		**Vitamin E** (**mg^b^**)		**Retinol** (***μ*g**)		***β*-Carotene** (***μ*g**)	
	**<100**	**⩾400**	**<9**	**⩾50**	**<400**	**⩾1800**	**<2400**	**⩾6000**
*NHS women*								
Median daily intake	86	694	8	187	285	2469	1907	7455
Age (years)	54	58	55	58	54	56	54	58
Postmenopausal (%)	57	73	62	76	59	73	57	77
Postmenopausal hormone user^c^ (%)	27	41	26	43	28	38	30	37
Nulliparous (%)	6	8	6	7	6	7	6	7
Body mass index (kg m^−2^)	25.7	25.1	25.6	25.1	25.4	25.2	25.6	25.2
Height (cm)	164	164	164	164	164	164	′164	164
								
*Nondietary risk factors*								
Painful burn or blisters during								
childhood after 2 h in sun (%)	15	15	14	15	15	15	15	15
6+ sunburns in lifetime (%)	55	58	53	59	56	57	57	55
3+ moles on left arm (%)	12	13	12	13	12	12	12	12
Red or blonde hair (%)	15	16	15	16	15	17	15	16
Parent or sibling with melanoma (%)	3	4	3	4	3	4	4	4
								
*NHS II women*								
Median daily intake	85	690	8	187	296	2375	1763	7721
Age (years)	39	41	39	42	40	39	40	42
Premenopausal (%)	88	86	89	84	87	88	89	87
Oral contraceptive user^d^ (%)	10	10	10	10	10	9	10	9
Nulliparous (%)	20	33	21	31	23	25	21	27
Body mass index (kg m^−2^)	26.0	24.9	25.4	24.7	25.6	25.0	25.9	25.2
Height (cm)	164	164	164	164	165	165	165	165
								
*Nondietary risk factors*								
Painful burn or blisters during								
childhood after 2 h in sun (%)	25	25	24	25	24	25	25	25
3+ sunburns in teen years (%)	28	28	26	27	28	27	27	28
5+ moles on lower legs (%)	21	22	21	23	21	22	22	21
Red or blonde hair (%)	19	22	20	22	19	22	20	21
Parent or sibling with melanoma (%)	5	6	5	6	5	5	5	5

a Values are means or percentages of the population within categories of nutrient intakes and are standardised to the age distribution of the study populations over follow-up (NHS 1984–1998; NHS II 1991–1999).

b mg of *α*-tocopherol equivalents.

c Percent postmenopausal hormone users is calculated among postmenopausal women only.

d Percent oral contraceptive users is calculated among premenopausal women only.

shows the characteristics of the study populations within low and high categories of vitamin C, vitamin E, retinol, and *β*-carotene intakes from foods plus supplements. For all nutrients, NHS women with higher intakes were older, and if postmenopausal, were more likely to use postmenopausal hormones. NHS II women with higher intakes were more likely to be nulliparous. In both cohorts, the nondietary risk factors for melanoma were not associated with nutrient intakes. Use of multivitamins and vitamin C and E supplements were high in both cohorts (53, 35, 36%, respectively, for NHS in 1998; 51, 27, 21%, respectively, for NHS II in 1999). Vitamin A (<5%) and *β*-carotene (<3%) supplement use were low in both cohorts.

We found no evidence that higher intakes of vitamin C, vitamin E, retinol, or *β*-carotene, the major carotenoid with provitamin A activity, are associated with a lower risk of melanoma (Table 2

**Table 2:** Relative risks (RRs) of melanoma by daily intakes of vitamin C, vitamin E, retinol, and *β*-carotene^a^

	**Cases**	**Person-years** (**thousands**)	**Age-adjusted^b^ RR** (**95% CI^d^**)	**Multivariate^c^ RR** (**95% CI^d^**)
*Vitamin C* (*mg*)				
Total^e^				
<110	60	315	1.00	1.00
110–159	89	363	1.26 (0.73–2.19)	1.21 (0.71–2.06)
160–219	78	312	1.31 (0.93–1.84)	1.21 (0.86–1.71)
220–399	81	323	1.31 (0.93–1.85)	1.19 (0.84–2.68)
⩾400	106	335	1.50 (0.78–2.89)	1.33 (0.74–2.38)
*P* for linear trend			0.27	0.53
				
Dietary^f^				
<90	57	325	1.00	1.00
90–114	73	307	1.32 (0.93–1.87)	1.27 (0.90–1.80)
115–139	74	322	1.29 (0.91–1.82)	1.24 (0.87–1.76)
140–174	112	353	1.72 (1.24–2.37)	1.62 (1.17–2.25)
⩾175	98	341	1.51 (1.08–2.11)	1.43 (1.02–2.00)
*P* for linear trend			0.04	0.05
				
*Vitamin E* (*mg^g^*)				
Total^e^				
<9.0	72	343	1.00	1.00
9.0–10.9	85	317	1.25 (0.77–2.04)	1.19 (0.75–1.90)
11.0–16.9	77	318	1.15 (0.83–1.59)	1.05 (0.76–1.45)
17.0–49.9	89	357	1.18 (0.68–2.07)	1.09 (0.64–1.83)
⩾50.0	91	313	1.25 (0.70–2.23)	1.11 (0.66–1.85)
*P* for linear trend			0.30	0.54
				
Dietary^f^				
<8.0	75	324	1.00	1.00
8.0–8.9	86	369	0.99 (0.73–1.36)	0.96 (0.70–1.31)
9.0–9.9	103	385	1.14 (0.85–1.54)	1.07 (0.79–1.44)
10.0–10.9	81	264	1.30 (0.95–1.78)	1.21 (0.88–1.66)
⩾11.0	69	306	0.93 (0.58–1.50)	0.88 (0.59–1.32)
*P* for linear trend			0.80	0.98
				
*Retinol (*μ*g)*				
Total^e^				
<400	87	322	1.00	1.00
400–699	71	351	0.75 (0.54–1.02)	0.72 (0.53–0.99)
700–1099	78	317	0.90 (0.66–1.23)	0.85 (0.63–1.16)
1100–1799	95	335	1.03 (0.77–1.38)	0.97 (0.72–1.30)
⩾1800	83	324	0.92 (0.68–1.24)	0.85 (0.63–1.16)
*P* for linear trend			0.80	0.52
				
Dietary^f^				
<300	79	309	1.00	1.00
300–424	76	332	0.90 (0.65–1.24)	0.88 (0.62–1.26)
425–599	95	368	1.03 (0.76–1.39)	0.99 (0.73–1.35)
600–849	74	317	0.91 (0.66–1.26)	0.87 (0.63–1.20)
⩾850	90	323	1.08 (0.75–1.56)	1.07 (0.74–1.55)
*P* for linear trend			0.96	0.99
				
*β-Carotene^h^* (*μg*)				
Total^5^				
<2400	56	320	1.00	1.00
2400–3399	111	358	1.75 (1.25–2.40)	1.67 (1.20–2.30)
3400–4399	73	320	1.24 (0.79–1.93)	1.17 (0.79–1.73)
4400–5999	94	331	1.54 (1.10–2.16)	1.44 (1.02–2.02)
⩾6000	80	320	1.33 (0.92–1.93)	1.22 (0.86–1.74)
*P* for linear trend			0.59	0.96

a Results from NHS and NHS II were pooled to obtain risk estimates for the combined cohorts. All *P*-values were >0.05 in tests for heterogeneity.

b Relative risks were adjusted for age and follow-up cycle. In addition, dietary vitamin C was adjusted for vitamin C supplement use; dietary vitamin E was adjusted for vitamin E supplement use; and dietary retinol was adjusted for multivitamin use and vitamin A supplement use.

c Relative risks were adjusted for the covariates listed above plus the following: skin reaction after 2h of sun exposure during childhood, number of sunburns over lifetime (NHS) or during teenage years (NHS II), number of moles on left arm (NHS) or on lower legs (NHS II), hair colour, family history of melanoma, state of residence, menopausal status, oral contraceptive use, postmenopausal hormone use, parity, height, and body mass index.

d 95% confidence interval.

e Total nutrient intakes are from foods plus supplements.

f Dietary nutrient intakes are from foods only.

g Milligram of *α*-tocopherol equivalents.

h *β*-carotene analyses for dietary intake were very similar to analyses of total intake because of low use of *β*-carotene supplements.

). Specific tocopherols (*α*-tocopherol, *β*-tocopherol, *δ*-tocopherol, *γ*-tocopherol) and other carotenoids, with or without provitamin A activity (*α*-carotene, lycopene, *β*-cryptoxanthin, lutein, and zeaxanthin), were also unrelated to risk (data not shown). Age-adjusted relative risks were only modestly attenuated when adjusted for the other melanoma risk factors.

Rather than the hypothesised inverse relation, we observed a positive association between dietary vitamin C and melanoma. Women with intakes ⩾175 mg day^−1^ had an RR of 1.43 (95% CI 1.02–2.00) compared to those with intakes <90 mg day^−1^. The dose – response association was also marginally significant (*P*=0.05). A significant linear relation remained when vitamin E, retinol, and *β*-carotene were added to the model (*P*=0.03).

We also examined nutrient supplements in relation to melanoma risk (Table 3

**Table 3:** Relative risks (RRs) of melanoma by use of vitamin supplements

	**Cases**	**Person-years (**thousands**)**	**Multivariate^b^ RR** (**95% CI^c^**)
*Multivitamins*			
Never	143	596	1.00
Past	83	302	0.96 (0.51–1.83)*
Current	185	700	1.02 (0.82–1.28)
			
*Vitamin C Supplements*			
Never	231	973	1.00
Past	51	209	0.97 (0.71–1.32)
Current	129	416	1.07 (0.64–1.77)*
			
*Vitamin E Supplements*			
Never	292	1189	1.00
Past	40	141	0.98 (0.70–1.38)
Current	79	268	1.02 (0.79–1.33)

* *P*<0.05 in test for heterogeneity of results from NHS and NHS II.

a Results from NHS and NHS II were pooled to obtain risk estimates for the combined cohorts.

b Relative risks were adjusted for the covariates listed in Table 2 footnote c. In addition, vitamin C supplement use was adjusted for vitamin C from food and vitamin E supplement use was adjusted for vitamin E from food.

c 95% confidence interval.

). Current use of multivitamins (a major source of retinol), and supplements of vitamins C and E were all unassociated with risk, and there was also no indication of benefit with longer duration of use. Vitamin A and *β*-carotene supplement use were too low to provide sufficient power for analysis.

Total fruits and vegetables or those with a high carotenoid content (⩾2000 IU serving^−1^) were also not associated with a lower risk of melanoma (Table 4

**Table 4:** Relative risks (RRs) of melanoma by daily servings of fruits and vegetables and by frequency of consumption of orange juice

	**Cases**	**Person-years (thousands)**	**Multivariate^b^ RR (95% CI^c^)**
*Total fruits*			
<1.0	62	349	1.00
1.0–1.4	58	274	1.16 (0.80–1.66)
1.5–2.4	151	524	1.42 (0.99–2.04)
2.5–3.4	83	303	1.37 (0.95–1.98)
⩾3.5	60	198	1.37 (0.86–2.20)
*P* for linear trend			0.22
			
*Total vegetables*			
<2.0	86	448	1.00
2.0–2.9	126	479	1.22 (0.92–1.62)
3.0–3.9	106	356	1.32 (0.97–1.78)
4.0–4.9	53	196	1.11 (0.76–1.62)
⩾5.0	43	169	1.01 (0.68–1.50)
*P* for linear trend			0.81
			
*Fruits and vegetables high in carotenoids*			
<0.25	50	263	1.00
0.25–0.49	116	447	1.30 (0.51–3.33)*
0.50–0.74	85	347	1.18 (0.49–2.85)*
0.75–0.99	68	236	1.40 (0.73–2.68)
⩾1.00	95	355	1.24 (0.67–2.29)
*P* for linear trend			0.78
			
*Fruits and vegetables high in vitamin C*			
<0.5	57	317	1.00
0.5–0.9	77	376	1.10 (0.77–1.55)
1.0–1.4	96	378	1.33 (0.94–1.87)
1.5–1.9	92	280	1.54 (1.06–2.22)
⩾2.0	92	297	1.43 (0.99–2.07)
*P* for linear trend			0.01
			
*Orange juice*^d^			
Never	39	228	1.00
1–3 month^−1^	70	364	1.09 (0.73–1.62)
1 week^−1^	44	197	1.31 (0.83–2.05)
2–6 week^−1^	203	799	1.44 (1.00–2.08)
⩾1 day^−1^	147	499	1.61 (0.92–2.84)
*P* for linear trend			0.008

* *P*<0.05 in test for heterogeneity of results from the NHS and NHS II.

a Results from NHS (1984–1998) and NHS II (1991–1999) were pooled to obtain risk estimates for the combined cohorts.

b Relative risks were adjusted for the covariates listed in Table 2 footnote c plus total energy intake, multivitamin use, and use of vitamin C, vitamin E, and *β*-carotene supplements.

c 95% confidence interval.

d Follow-up for NHS is 1980 – 1998.

) (see Feskanich *et al* (2000) for a description of the fruits and vegetables). However, consumption of fruits and vegetables high in vitamin C (⩾30 mg serving^−1^) was significantly associated with an increasing risk (*P* for linear trend=0.01). Orange juice was the major contributor to dietary vitamin C (about 20% in both cohorts), and ⩾1 serving day^−1^ was associated with an elevated risk of melanoma when compared to no orange juice consumption (RR=1.61, 95% CI 0.92–2.84, *P* for linear trend=0.008). In the NHS cohort, women recalled their frequency of orange juice consumption during high school, and this too was associated with an increased risk of melanoma (RR= 1.59, 95% CI 1.17–2.18 for ⩾1 serving day^−1^ *vs* <1 serving week^−1^). When analysed in the same model, significant positive dose–response relations were observed for both adult (*P*=0.01) and high school (*P*=0.04) orange juice consumption. In an analysis of consistent intake over time, women who reported that they consumed ⩾1 serving day^−1^ of orange juice in both high school and as an adult had an RR of 2.32 (95% CI 1.36–4.00) compared to those who reported <1 serving week^−1^ in both time periods.

Since dietary vitamin C was not expected to be a risk factor for melanoma, we speculated that the positive associations might be because of greater sun exposure among women with higher vitamin C intakes. The highest incidences of melanoma were found among the NHS women who lived in Florida (43 out of 100 000 year^−1^) and California (31 out of 100 000 year^−1^), states where excessive sun exposure is more likely. However, their dietary intakes of vitamin C were not higher than the rest of the cohort and excluding them from analyses did not change results from those in Table 2 that were adjusted for state of residence.

Since nondietary determinants of melanoma contribute significantly to risk, we examined associations between vitamins A, C, E, and melanoma separately among women at higher and lower risk based upon nondietary factors. A total risk score was calculated using cohort-derived RRs associated with each of the five nondietary factors listed in Table 1. The lowest score of 5 was assigned to women with the lowest relative risk (RR=1.0) for all factors; the highest risk scores were 11.9 in NHS and 14.3 in NHS II. Categories of low, middle, and high risk were defined to ensure approximately equal numbers of cases in each category. Table 5

**Table 5:** Relative risks (RR) of melanoma by daily intakes of dietary vitamin C and total retinol, stratified by risk based on nondietary factors

	**Low risk**			**Middle risk**			**High risk**			
	**Cases**	**P-Y^c^**	**Multivariate^b^ RR**(**95% CI^d^**)	**Cases**	**P-Y^c^**	**Multivariate^b^ RR**(**95% CI^d^**)	**Cases**	**P-Y^c^**	**Multivariate^b^ RR**(**95% CI^d^**)	**Interaction^e^**
*Dietary*										
*Vitamin C* (*mg*)										*P*=0.12
<90	22	167	1.00	12	75	1.00	19	40	1.00	
90–114	24	158	1.15 (0.64–2.07)	25	74	2.10 (1.01–4.37)	16	39	0.82 (0.41–1.64)	
115–139	17	163	0.78 (0.41–1.49)	27	81	2.34 (1.15–4.76)	21	41	1.12 (0.60–2.12)	
140–174	32	179	1.29 (0.74–2.25)	34	90	2.25 (1.10–4.62)	37	44	1.76 (0.99–3.13)	
⩾175	29	172	1.16 (0.65–2.06)	25	85	1.55 (0.41–5.82)	35	42	1.68 (0.93–3.02)	
*P* for linear trend			0.68			0.18			0.01	
										
*Total*										
*Retinol* (*μg*)										*P*=0.001
<400	38	162	1.00	16	74	1.00	19	38	1.00	
400–699	22	186	0.51 (0.30–0.86)	20	86	0.99 (0.50–1.94)	26	44	1.07 (0.58–1.95)	
700–1099	24	162	0.61 (0.28–1.35)	28	79	1.47 (0.78–2.77)	21	41	0.89 (0.47–1.69)	
1100–1799	23	168	0.50 (0.29–0.85)	30	83	1.56 (0.84–2.89)	33	43	1.39 (0.78–2.48)	
⩾1800	17	160	0.39 (0.22–0.71)	29	82	1.50 (0.81–2.81)	29	40	1.36 (0.76–2.44)	
*P* for linear trend			0.01			0.16			0.46	

a Results from NHS (1984–1998) and NHS II (1991–1999) were pooled to obtain risk estimates for the combined cohorts. All *P*-values were >0.05 in tests for heterogeneity. See text for description of risk score.

b Relative risks were adjusted for the covariates listed in Table 2 footnote c.

c Person-years of follow-up (thousands).

d 95% confidence interval.

e *P*-value for interaction between risk score and nutrient intake.

shows results for dietary vitamin C and total retinol, the only nutrients which exhibited possible interactions with the risk score. For dietary vitamin C, a significant positive association was found only among women in the high-risk category (*P* for linear trend=0.01), although the test for interaction was not significant (*P*=0.12). For total retinol, a significant inverse association was observed in the low-nondietary-risk category. An RR of 0.39 (95% CI 0.22–0.71) was associated with a total retinol intake of ⩾1800 *μ*g day^−1^ when compared to <400 *μ*g day^−1^ (*P* for linear trend=0.01) and there was a significant interaction between total retinol and risk score (*P*=0.001).

## DISCUSSION

In this prospective study within two large cohorts of Caucasian women 25–77 years of age, we found little evidence that vitamin C, vitamin E, individual tocopherols, retinol, *β*-carotene, or other carotenoids are associated with a lower risk of invasive melanoma. The only inverse association that we observed was for total retinol intake when limited to women who were otherwise at low risk of melanoma based on nondietary factors. Although not an antioxidant, retinol is essential for regulating cell differentiation, and it is possible that it may only exert a notable effect without the overwhelming influence of other major risk factors. There is little prior research to support our finding. Breslow *et al* (1995) reported an RR of 0.4 when comparing extreme tertiles of plasma retinol levels, although there were only 30 melanoma cases in this analysis and results were not significant. Other case–control studies did not find an association between plasma (Stryker *et al*, 1990; Knekt *et al*, 1991) or dietary (Stryker *et al*, 1990; Kirkpatrick *et al*, 1994) levels of retinol and risk of melanoma. It is possible that our observed inverse association for total retinol intake among the low-risk women is a chance finding, but even if real, we would not recommend high retinol intakes for women at such low risk of melanoma since higher intakes have been associated with increased risk of hip fracture among postmenopausal women (Feskanich *et al*, 2002).

An unexpected finding was the increased risk of melanoma with higher intakes of vitamin C from food, and more frequent consumption of fruits and vegetables high in vitamin C, particularly orange juice. It is possible that this, too, is a chance finding. However, given that this association was strongest among the higher risk sun-sensitive women, it is also possible that a photosensitising compound in these foods is responsible for the increased risk; vitamin C is unlikely to be responsible because supplement users were not at higher risk. Furocoumarins are naturally produced in fruits and vegetables in response to physical damage, fungal infection, and attack by pests, and they exhibit phototoxic effects because of their capacity to absorb photons and then release the energy to cause cellular damage (Gasparro *et al*, 1997). Plants of the umbelliferae family, such as parsnips, celery, fennel, and parsley, have a high furocoumarin content, and dermatologic reactions have been observed with ingestion of large quantities and subsequent exposure to UV radiation (Ljunggren, 1990; Puig and Demaragas, 1994). Some types of limes (Nigg *et al*, 1993) and oranges (Joint Food Safety and Standards Group, Food Standard Agency, UK, 1993) also contain appreciable quantities of furocoumarins and have been noted to trigger dermatologic reactions when accompanied by sun exposure (Gross *et al*, 1987; Wagner *et al*, 2002). Psoralen is a furocoumarin that has long been used with UV A radiation to treat psoriasis and other skin conditions (Parrish *et al*, 1974) and more recently has been associated with higher risk of squamous cell cancer (Lever and Farr, 1994; Stern *et al*, 1998) and melanoma (Stern *et al*, 1997). Whether the amounts of furocoumarins in orange juice, when ingested over many years, are sufficient to affect risk of melanoma in susceptible individuals is unclear.

The major strength of this study is the prospective design; previous research has been limited to retrospective studies. Other strengths are the wide age distribution of women, the repeated measures of diet and supplement use, and the ability to control for several major risk factors for melanoma. Although we lacked lifetime information on time spent in the sun, the risk factor data that we did collect were unrelated to diet and therefore did not confound results.

In conclusion, higher intakes of vitamins C, E, and A were generally not associated with lower risks of melanoma in Caucasian women, although retinol may be protective among women who are otherwise at low risk of disease. Certain foods, such as orange juice, may be associated with an increased risk of melanoma, but further research is needed to confirm this finding and to identify the responsible constituents in these foods.
